# Recovery of Polyphenolic Fraction from Arabica Coffee Pulp and Its Antifungal Applications

**DOI:** 10.3390/plants10071422

**Published:** 2021-07-12

**Authors:** Jiraporn Sangta, Malaiporn Wongkaew, Tibet Tangpao, Patchareeya Withee, Sukanya Haituk, Chaiwat Arjin, Korawan Sringarm, Surat Hongsibsong, Kunrunya Sutan, Tonapha Pusadee, Sarana Rose Sommano, Ratchadawan Cheewangkoon

**Affiliations:** 1 Interdisciplinary Program in Biotechnology, Graduate School, Chiang Mai University, Chiang Mai 50200, Thailand; Jiraporn_sangta@cmu.ac.th; 2 Plant Bioactive Compound Laboratory, Faculty of Agriculture, Chiang Mai University, Chiang Mai 50200, Thailand; malaiporn@rmutl.ac.th (M.W.); tibet_tangpao@cmu.ac.th (T.T.); 3 Program of Food Production and Innovation, Faculty of Integrated Science and Technology, Rajamangala University of Technology Lanna, Chiang Mai 50300, Thailand; 4 Department of Plant and Soil Sciences, Faculty of Agriculture, Chiang Mai University, Chiang Mai 50200, Thailand; tonapha.p@cmu.ac.th; 5 Department of Entomology and Plant Pathology, Faculty of Agriculture, Chiang Mai University, Chiang Mai 50200, Thailand; patchareeya_withee@cmu.ac.th (P.W.); sukanya_hai@cmu.ac.th (S.H.); 6 Department of Animal and Aquatic Science, Faculty of Agriculture, Chiang Mai University, Chiang Mai 50200, Thailand; chaiwat_arjin@cmu.ac.th (C.A.); korawan.s@cmu.ac.th (K.S.); 7 School of Health Science Research, Chiang Mai University, Chiang Mai 50200, Thailand; surat.hongsibsong@cmu.ac.th (S.H.); kunrunya.s@cmu.ac.th (K.S.); 8 Research Institute for Health Science, Chiang Mai University, Chiang Mai 50200, Thailand; 9 Innovative Agriculture Research Center, Faculty of Agriculture, Chiang Mai University, Chiang Mai 50200, Thailand

**Keywords:** antifungal, antioxidant, bioactive compounds, extracts, flavonoids, losses, phenolic, value-adding components

## Abstract

Coffee pulp is one of the most underutilised by-products from coffee processing. For coffee growers, disposing of this agro-industrial biomass has become one of the most difficult challenges. This study utilised this potential biomass as raw material for polyphenolic antifungal agents. First, the proportion of biomass was obtained from the Arabica green bean processing. The yield of by-products was recorded, and the high-potency biomass was serially extracted with organic solvents for the polyphenol fraction. Quantification of the polyphenols was performed by High Performance Liquid Chromatography (HPLC), then further confirmed by mass spectrometry modes of the liquid chromatography–quadrupole time-of-flight (QTOF). Then, the fraction was used to test antifungal activities against *Alternaria brassicicola*, *Pestalotiopsis* sp. and *Paramyrothecium breviseta*. The results illustrated that caffeic acid and epigallocatechin gallate represented in the polyphenol fraction actively inhibited these fungi with an inhibitory concentration (IC50) of 0.09, 0.31 and 0.14, respectively. This study is also the first report on the alternative use of natural biocontrol agent of *P. breviseta*, the pathogen causing leaf spot in the Arabica coffee.

## 1. Introduction

Coffee is a beverage cash crop that is widely cultivated in the tropicals, especially in the Americas, Africa, and Asia with global production reaching 10.5 million tons per year [1]. *Coffea arabica* L. is cultivated mainly for the premium quality that the selling price is twice as much as other varieties [2,3]. As such, the production volume of Arabica coffee is up to 60–65% and the demand is expected to increase by ca. 10% per year [4,5]. During the coffee processing, the biological losses are accounted for up to 40–45% and the losses include pulp, husk, parchment, silver skin and spent coffee grounds [6]. Coffee pulp is weighed as high as 29% of the total dried weight which is disposed of as waste and has become an environmental pollution that incurs a high cost of management [7]. Therefore, considering their availability, which is practically of low cost, attempts have been made in order to value-add this agro-industrial biomass through the recovery of bioactive components [6,7,8,9,10].

The coffee pulp contains carbohydrates, proteins, fibres, fats, and antioxidants such as phenolic compounds, chlorogenic acid, epicatechin [11,12] and caffeine [13]. Plant phenolic compounds are known as effective pathogenic fungi inhibiting agents [14,15,16]. The possible modes of actions include but are not limited to, toxic effects, inducing cell apoptosis, inhibition of hypha development, inhibition of biofilm formation and disruption of cell membrane integrity [14,15,16]. However, the studies on the utilisation of coffee pulp extract to use against plant pathogenic fungi were only a few. Presently, *Alternaria brassicicola* is the major pest that causes pre-postharvest diseases of various vegetables such as cabbages and kales [17,18]. *Pestalotiopsis* sp. induced postharvest diseases in tropical fruits, cut flowers and a new type of leaf fall of para rubber [19,20,21,22]. Furthermore, *Paramyrothecium breviseta* was recently isolated from the leaf spot of the Arabica coffee [23,24,25]. To fill in the gap mentioned above as to increase the value of the coffee pulp biomass, the objectives of this research are to investigate types of biomass and losses during Arabica coffee processing and to serially extract the Arabica coffee pulp and analyse the chemical compositions. The fraction was then used as plant pathogenic antifungal agents. The outcome of this study would ideally support the global sustainable development goals (SDGs) by providing an alternative way to minimise the volume of non-renewable materials, thereby reducing the cost of management and carbon footprint attached to coffee production.

## 2. Results and Discussion

### 2.1. The Phytochemical Profiles of Coffee Pulp Powder

**Figure 1:**
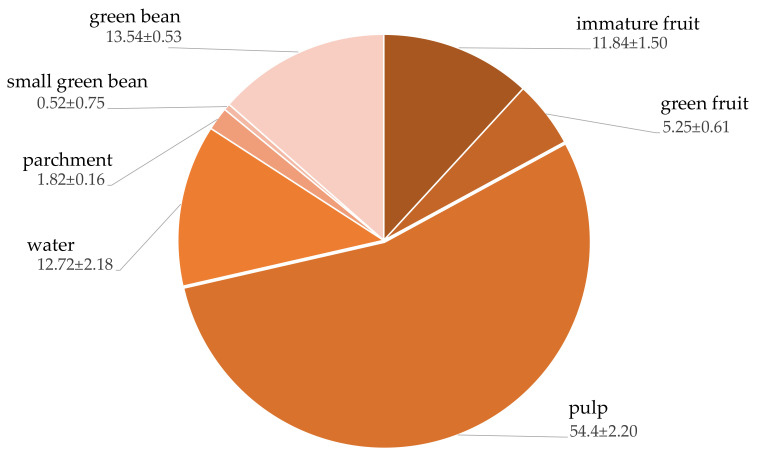
Losses and types of by-products from Arabica coffee processing.

From the initial processing step, the data indicated that the high-quality green bean yielded only 13% of the total harvesting weight while the others remained as processing by-products. The major by-products consisted of 54% of pulp, 12% of immature fruit and 5% of green fruit (Figure 1).

In Table 1, carbohydrate was the major constituent followed by crude fibre, crude protein, crude fat and ash, respectively. The phytochemical compositions were not apparently different in both steps, except for the contents of crude fat and crude fibre. Carbohydrate is a dominant nutrient in the coffee pulp which is accounted for in the range 35.0–66.0% [26]. The carbohydrate compositions comprised reducing sugars (5.4%) and a high content of pectin (20.5%) [27]. Pectin was possibly separated from the pulp and precipitated from other components by methanol, thus a slight reduction in the content was observed.

The crude fibre content of the coffee pulp powder was ~18% and remained stable either before or after extraction. In other works, dried coffee pulp obtained from the residue of wet processing is widely used as a source of dietary fibre (33.6%) [28,29]. The dietary fibre in the soluble type of carbohydrate is run off during the extraction process, particularly when a highly polar solvent is used [30].

The protein content of the coffee pulp before and after methanolic extraction was ~12%, which is in line with the studies of Ameca et al. [31] and Setyobudi et al. [26]. The elevating content of protein in the pulp may involve the enzyme excretion (polygalacturonase, pectin methylesterase and galactosidase) for pectin modification during the maturity stage [32].

The reduction of crude fat contents in the BCF (4.1%) to the ACF (1.2%) was notably distinct. The major component of fat in coffee is unsaturated fatty acids (such as linoleic acid) [33], which are highly dissolved in methanol, based on the principle of “like dissolves like” [34]. The content of crude fat was somewhat low when compared to other constituents. Other studies reported that fat content was in the range of 0.8–7.0% [26]. Mostly, fat content in coffee was in the form of polyunsaturated and saturated fatty acids [33]. Ash content in coffee pulp was the lowest ~0.30% in both types of raw materials. These values were much lower than what had been reported by Ameca et al. [31] and Figueroa and Mendoza [35] at 7.0%. Generally, ash referred to minerals, as well as both micro and macronutrients [36].

The yield of the extracts obtained from methanolic extraction (6.6%) was higher than that of dichloromethane (2.3%) extraction. Based on the polarity of the solvent, where methanol has greater polarity than that of dichloromethane, as the major important substances in the pulp are the polar substances, especially those of the polyphenols [37], it is evident that the recovery of the phenolic compounds was dependent on the solvent used and its polarity [38]. Polyphenols are often soluble in organic solvents that are less polar than water. Effective extraction of plant material depends on the choices of the solvent, extracting temperatures, and mechanical agitation to maximise the polyphenol recovery [39].

Polyphenols, secondary metabolites, are categorised into two classes; flavonoids and phenolic acids [40]. The amount of total flavonoid recovered from the coffee pulp extract was higher than that of total phenolic contents approximately two times by different means of standards. These contents in the dichloromethane fraction, however, were not detected (Table 1). This was comparable to Delgado et al. [41]. Rodríguez-Carpena et al. [42] described that the recovery of the polyphenols from plant materials is affected by the solubility of the phenolic compounds in the solvent of choice. Consequently, solvent polarity plays a key role in increasing phenolic solubility [43]. Geremu et al. [37] reported that the concentration of the total polyphenols was the highest when methanol was used followed by acetone and ethanol, respectively. Therefore, in substantial studies, only the methanol fraction was used.

The antioxidant activities of the extracts obtained from methanol and dichloromethane were also evaluated using DPPH and ABTS assays. The methanol extract provided greater efficiency in both assays than dichloromethane. The same result was also reported by Geremu et al. [37], by which the methanolic extract gave the highest scavenging activity (17.3–70.2%) when comparing ethanol to the acetone extracts. This corresponds with the high polyphenol content that is able to scavenge free radicals with their hydroxyl groups. The finding is in agreement with Haifeng et al. [44] who reported higher polyphenol content along with high antioxidant activity. Therefore, the polyphenol content of plants may contribute directly to their antioxidant potency [45].

### 2.2. Quantitative Polyphenol Analyses

Table 2 illustrates the polyphenol compositions (flavonoid and phenolic compounds) in the coffee pulp. Among all others, epigallocatechin gallate was dominant (32.0 mg/g extract) and caffeic acid (68.0 mg/g extract) for the flavonoids and non-flavonoids, respectively. The flavonoids are further classified into flavones (apigenin), flavanones (naringenin), flavonols (quercetin), flavanols (catechin), isoflavones (daidzein), as well as the phenolic acids are grouped into hydroxybenzoic (gallic acid, protocatechuic acid) and hydroxycinnamic acids (coumaric acid, caffeic acid) [46]. The content of non-flavonoids (caffeic acid, caffeine, p-coumaric acid, rosmarinic acid, o-coumaric acid, quercetin, gallic acid) were greater than that of the flavonoid contents (epigallocatechin gallate, naringenin, epicatechin gallate, catechin, gallocatechin gallate). The highest content of flavonoid and non-flavonoid compounds were epigallocatechin gallate (31.8%) and caffeic acid (68.1%), respectively. Heeger et al. [47] reported that chlorogenic acid, gallic acid, protocatechuic acid and rutin are the most prominent compounds identified in the coffee pulp extracts (>80.0% of polyphenol content). The presence of chlorogenic acid (42.2%), epicatechin (21.6%), rutin (2.1%), catechin (2.2%), ferulic acid (1.0%) and protocatechuic acid (1.6%) has also been found in the pulp extracted with 80.0% methanol [48].

**Figure 2:**
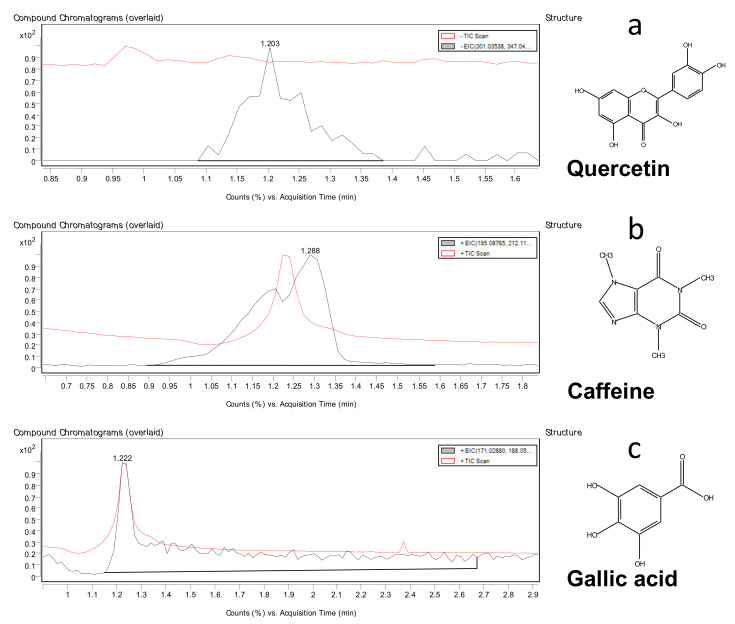
The quadrupole time-of-flight mass chromatograms with the EIC and +TIC scan of the polyphenols in crude methanol fraction of the coffee pulp powder with the chemical structures (**a**) quercetin, (**b**) caffeine and (**c**) gallic acid.

The presence of the polyphenols was confirmed by the scanning modes -ESI+ and LC-ESI- modes by the protocol set earlier in our previous work as shown in Table 3 [49]. Among the 12 quantified compounds derived from the HPLC standard curves of the polyphenol and catechin, we were only able to confirm quercetin, gallic acid and caffeine with the *m*/*z* values of 361.0, 171.0 and 195.0, respectively by the QTOF-MS possibly due to the impurity of the crude methanolic fraction. These compounds were selected at a minimum of 80% matching score. The chemical structures of the confirmed compounds are illustrated in Figure 2. Low signal performance of the spectrometry was also problematic for crude methanolic extract in our previous study [50]. We recommended that for further structure elucidation the purification step should be undertaken.

### 2.3. Antifungal Activities

**Figure 3:**
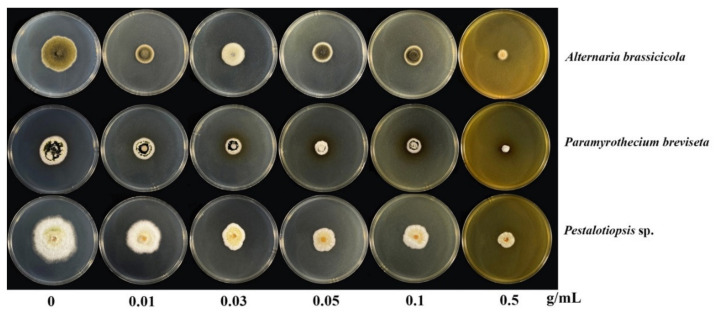
Inhibitory effect (inhibition zone, mm) after 7 days of inoculation of the crude methanolic fraction of coffee pulp powder against 7 days mycelium plug of *Alternaria bassicicola* (CRC152), *Pestalotiopsis* sp. (CRC151) and *Paramyrothecium breviseta* (CRC12). The concentrations of poison food were prepared at 0, 0.01, 0.03, 0.05, 0.1 and 0.5 g/mL extract in the potato dextrose agar.

The results of the antifungal bioassay of the crude methanolic coffee pulp extract at different concentrations are given in Table 4 with their comparative effectiveness as shown in Figure 3. The results indicated that at 0.5% concentration, the methanolic extract inhibited 78.0% growth against *P. breviseta*, 71.0% against *A. brassicicola* and the lowest at 62.0% inhibition against *Pestalotiopsis* sp. The result convinces us that the coffee pulp methanolic extract is able to accomplish antifungal activity in vitro and, more importantly, the inhibition increases with the increasing extract concentrations [51]. Gupta et al. [51] reported that two botanical extracts *viz. Azadirachta indica*, *Capsicum annum* were found to be highly effective against *A. brassicicola* at both 15.0% and 25.0% concentrations and the mycelial inhibition was recorded at 68.0% and 25.0% concentrations.

The 50.0% inhibitory concentration of the mycelial growth of *A. brassicicola*, *Pestalotiopsis* sp. and *P. breviseta* was 0.09, 0.31 and 0.14 g/mL, respectively (Table 4). Chen et al. [52] reported a minimum concentration of crude extract that was able to inhibit conidial germination of *A. solani* at the lowest (0.19 mg) concentration after a 72 h incubation period. However, the chemical profile of the extract was not reported. Phytochemical extracts are known to exhibit antimicrobial activity from a wide variety of secondary metabolite compositions [49,53]. Crude extract from plants might be a good candidate for an antifungal agent. *Pestalotiopsis* spp. can cause fruit rot in Chinese olives [54,55]. In this study, *Pestalotiopsis* sp. was isolated from leaf spot disease of rose apple, however, the effective dose of the coffee pulp extract was the highest. We believe that as an endophyte pathogen, it is able to repel natural products synthesised within the plants per se, thereby requiring a higher concentration for fungal inhibition. Such a mechanism has been described previously [52]. Finally, the report on antifungal effects of *P. breviseta* has not been documented anywhere.

**Figure 4:**
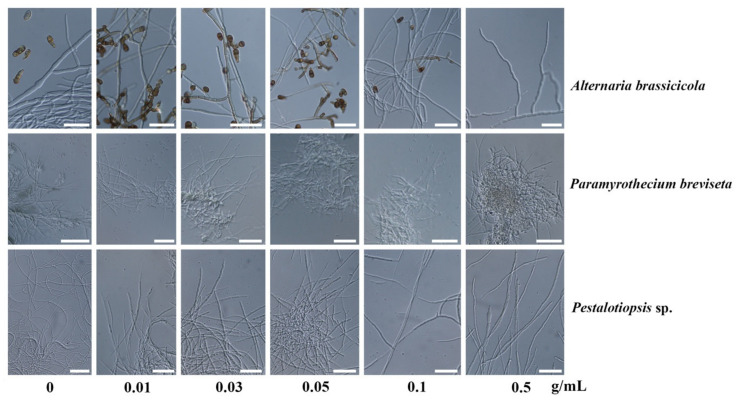
Effects of the crude methanolic fraction of coffee pulp powder on the fungal morphology of *Alternaria brassicicola* (CRC152), *Pestalotiopsis* sp. (CRC151) and *Paramyrothecium* *breviseta* (CRC12) after culturing on the poison food after 7 days of inoculation. The concentrations of poison food were prepared at 0, 0.01, 0.03, 0.05, 0.1 and 0.5 g/mL extract in the potato dextrose agar.

The numbers of spore counts in *A. brassicicola* and *P. breviseta* decreased with the increasing concentrations of the extract in the poison food until reaching the maximum concentration (Figure 4). The sporulation of the fungi could be induced by environmental stress including lack of nutrients, the resistance of the host tissues and UV light [56,57,58,59]. Plant natural products could initiate fungal cell wall stress, thereby inducing sporulation and inhibiting fungal growth [60]. The concentration of the extracts in the potato dextrose agar, however, did not affect spore morphology as described by spore width and length for *P. breviseta.* Indeed, the result indicated the morphology alteration at the concentration of 0.1 mg/mL in the *A. brassicicola*. The mycelial morphology did not illustrate many changes in the poison food. It is possible that light microscopy would not be able to get clear photographs of mycelium morphology. The aberrant mycelium morphology may necessitate examination with a scanning electron microscope (SEM).

### 2.4. Chemometric Relations

**Figure 5:**
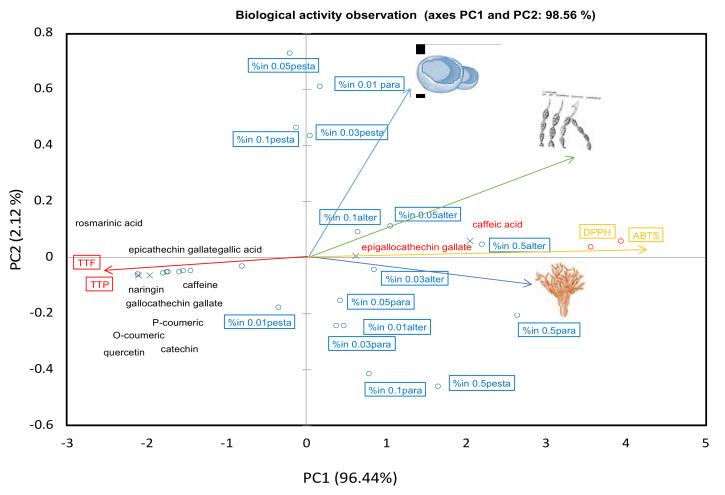
Chemometric score plot of the antifungal activities described as percentage of inhibition (%in) of *Alternaria brassicicola* (CRC152), *Pestalotiopsis* sp. (CRC151) and *Paramyrothecium* *breviseta* (CRC12) in different concentrations of crude methanolic extracts of coffee pulp 0, 0.01, 0.03, 0.05, 0.1 and 0.5 g/mL. Abbreviations; alter = *Alternaria* spp.; pesta = *Pestalotiopsis* spp.; para = *Paramyrothecium* spp.; TTF = total flavonoid content; TTP = total phenolic content.

The chemometric multivariate has been used to comprehend the relationships between bioactive ingredients and biological activities by many studies [53,54,55]. The score plot relationship of the polyphenols and the biological activities are illustrated in Figure 5. All variations were well distributed across the plot which accounted for 96.4% in the PC1 and 2.0% in the PC2. As depicted in the Figure, the biological functional properties as described by the antioxidant activities (either by the DPPH and ABTS assays) and antifungal activities against all fungal strains clustered together (marked in yellow and blue). These were projected closely to caffeic acid and epigallocatechin gallate. In contrast, the total phenol and flavonoid displayed among the other polyphenols were projected at the opposite end of the score plot. We were then convinced that the caffeic acid and epigallocatechin gallate were responsible for the antifungal properties. Caffeic acid induces the pathogenic lipolytic enzymes which disintegrate the cell membranes of the fungus, thereby causing cell leakage. Additionally, it can directly inhibit protein synthesis in pathogen cells [51,56]. Thus, caffeic acid and its derivatives have been used against many plant-fungal pathogens such as *Aspergillus niger*, *Fusarium graminearum* and *A. alternata* that cause food spoilage, post-harvest browning and plant head white diseases [57,58]. Epigallocatechin gallate also interferes with fungal cell wall integrity by directly binding to peptidoglycan which has affinities toward various cell wall components [59]. Among all others, these coffee polyphenols could also inhibit mycelial growth, inducing the production of H_2_O_2_ leading to lipid peroxidation, and the leakage of K+, soluble protein and soluble sugars that are responsible for the increased cell membrane permeability [60].

## 3. Materials and Methods

### 3.1. Raw Material

Fruits of the Arabica coffee (*C. arabica* L.) in a commercial harvesting stage (~80% red of the overall skin colour) were hand picked from Khun Changkhian Highland Agricultural Research and Training Station, Faculty of Agriculture, Chiang Mai University (18.840093525158775, 98.89823401100753) in January 2021. The coffee cherry was transported to the coffee processing site immediately after harvest, which they were then cleaned by floating in tap water prior to wet processing [61]. The defective and green fruits were then eliminated by this step. Losses (%) were recorded as biomass weight to the total mass of raw materials. The losses of coffee processing consisted of floating and green fruits, pulp, wet parchment and parchment. The coffee pulp was then dehydrated by sun-drying for 2 weeks followed by hot air drying until constant moisture content was reached (4.0–6.0%). The dried material was ground to a fine powder using a high-speed food processor for subsequent uses [62].

### 3.2. Microorganisms

*Alternaria brassicicola* (CRC152), *Pestalotiopsis* sp. (CRC151) and *Paramyrothecium* *breviseta* (CRC12), obtained from the fungus collections of the Department of Entomology and Plant Pathology, Faculty of Agriculture, Chiang Mai University were used for antifungal activity tests. They were originally isolated from lettuce (*Brassica rapa* subsp. *pekinensis*), oil palm (*Elaeis guineensis* L.) and coffee (*C. arabica* L.), respectively [63]. Morphological characteristics of the fungus were identified using a Stemi 305 Zeiss stereo microscope and Axiovision Zeiss Scope-A1 microscope. The isolates were submerged in 10.0% glycerol and kept at 4 °C before use. All isolates were activated on the prepared potato dextrose agar (PDA) and incubated at room temperature (28 ± 2 °C) for 7 days before being used [64].

### 3.3. Proximate Analyses

Coffee pulp powder was analysed for proximate compositions according to the methods of the Association of Official Analytical Chemists (AOAC, 2000) [62]. Crude fibre content was analysed using the fibre analyser (Fibertherm FT12, Gerharadt, Germany).

### 3.4. Polyphenolic Fractions

The extraction was according to the serial extraction as described in Wisetkomolmat et al. [65] with some modifications. One hundred grams of the coffee pulp (CF) was first extracted with 400 mL of 95% dichloromethane for 24 h at room temperature to remove compounds of low polarity such as fatty acids. The extract was then separated through filter paper Whatman No.1. The filtrate was concentrated using a rotary evaporator at 40.0 °C and used as the crude dichloromethane fraction. After that, the remaining CF was extracted with 400 mL of 80% methanol, filtered, and concentrated as the crude methanol fraction. Yields of those fractions were recorded, accordingly. Also, for comparison, the proximate contents of the biomass after the serial extractions were also examined.

### 3.5. Quantitative Polyphenol Analyses

#### 3.5.1. Total Phenolic Content

As described by Sunanta et al. [66], the extract (30 µL) was mixed with 60 µL of Folin–Ciocalteu reagent and then neutralized with 210 µL of 6.0% *w*/*v* saturated sodium bicarbonate and kept at room temperature in the darkness for 2 h. The absorbance reading was taken at a wavelength of 725 nm by UV-Vis spectrophotometer (SPECTROstar, BMG LABTECH, Offenburg, Germany). The calibration standard was prepared using different concentrations of gallic acid (10–200 mg/mL). The total phenolic content was expressed as milligram gallic acid equivalents per gram of dried sample.

#### 3.5.2. Total Flavonoid Content

The content of total flavonoid was determined using the modified method of Sunanta et al. [66]. The methanolic crude extract (25 µL) was mixed with 125 µL of distilled water and 7.5 µL of 5.0% NaNO_2_ solution was then added. The mixture was left to stand at room temperature for 5 min, thereafter 15 µL of 10.0% AlCl_3_·6H_2_O was added and incubated for 6 min. After that, 50 µL of 1 M of NaOH and 27.5 µL distilled water was subsequently added. The absorbance of the test solution was measured at a wavelength of 510 nm using the UV-Vis spectrophotometer. The catechin calibration standard was prepared at different concentrations (30–300 mg/mL). The total flavonoid content was expressed as milligram catechin equivalents per gram of dried sample.

### 3.6. Antioxidant Activities

#### 3.6.1. DPPH^•^ Radical Scavenging Activity

The free radical-scavenging activity was determined using the method described by Sunanta et al. [66]. Twenty-five microliters of the extract were added with 250 µL of 0.2 mM DPPH (2,2-diphenyl-1-picrylhydrazyl) and incubated in the darkness at room temperature for 30 min. The absorbance was measured at a wavelength of 510 nm using the UV-Vis spectrophotometer. The DPPH radical scavenging was calculated using the following equation;

DPPH radical scavenging activity (%) = [(Abs_control_ − Abs_sample_)]/(Abs_control)_] × 100 (1)

where Abs_control_ is the absorbance of DPPH radical mixed with methanol; Abs_sample_ is the absorbance of DPPH radical reacted with sample extract/standard.

#### 3.6.2. Total Antioxidant Activity by ABTS^•+^ Radical Cation Decolourization Assay

For ABTS [2,2-azino-bis-(3-ethylbenzothiazoline-6-sulfonic acid)] assay, the method of Adedapo et al. [67] was adjusted briefly. The working solution was prepared by mixing the two stock solutions including 7.0 mM ABTS solution and 2.45 mM potassium persulfate solution. The solutions were mixed in equal quantities and the solution was allowed to react in the darkness at room temperature for 12–16 h. The ABTS solution was prepared by diluting 1.0 mL ABTS with 60.0 mL of 80.0% methanol to obtain an absorbance of 0.7 ± 0.02 units at 734 nm. Thereafter, 10 µL of methanol extract and 200 µL of ABTS working solution were pipetted into the microplate well, shaken and incubated at room temperature for 30 min. The absorbance was then taken at 734 nm. The ABTS scavenging capacity of the extract was calculated by the following equation;

ABTS radical scavenging activity (%) = [(Abs_control_ − Abs_sample_)]/(Abs_control_)] × 100 (2)

where Abs_control_ is the absorbance of ABTS radical mixed with 80% methanol; Abs_sample_ is the absorbance of ABTS radical reacted with sample extract/standard.

### 3.7. Antifungal Activities

The methanol extract was dissolved in 95% methanol at different concentrations (0.01, 0.03, 0.05, 0.1 and 0.5 g/mL). For the preparation of the poison food, 1 mL of each sample was supplemented in a sterilised potato dextrose agar (PDA) and poured onto the petri dish. The active fungal mycelium (plug) cultivated for 7 days (6 mm diameter) was placed into the centre of the media and incubated at room temperature (28 ± 2 °C) for 7 days. Growth development of fungal mycelium was measured and compared with negative control (without the extract supplementation). Images of spore and mycelium were taken by a Canon 6D camera connected with an Axiovision Zeiss Scope-A1 microscope. All measurements were made using the Tarosoft^®^ Image Framework program v.0.9.0.7. The percentage of growth inhibition of the fungi was calculated using the following equation;

Percentage inhibition (%) = [(R1 − R2)/R1] × 100(3)

where R1 is the colony radius in the control plate and R2 is the radial growth of the pathogen in the presence of plant extract.

### 3.8. Quantitative Analysis of Phenolic and Flavonoid

The methanol fraction was dissolved in 95.0% methanol to a final concentration of 1 mg/mL and analysed for the polyphenol contents using a High-Performance Liquid Chromatography analysis (HPLC) (Shimadzu, Kyoto, Japan) with an automatic injection (SIL-20ACHT), diode array detection (CTO-20AC), pump (LC-20AD) and automatic control (CBM-20A). The running protocol consisted of two conditions. The first condition was according to the modified method of Lux et al. [68]. Reverse-phase column chromatography was performed using an Ultra Aqueous C18 (250 × 4.6 mm, 5 μm) (RESTEK, Bellefonte, PA, USA). The mobile phase consisted of the mixtures of A and B, where mixture A contained formic acid and distilled water with a ratio of 5:95 and mixture B included acetonitrile (ACN) formic acid and distilled water with a ratio of 85:50:10. The gradient elution started at a flow rate of 1 mL/min and the injection volume was 10 μL. The initial condition was 80% A in 4 min and decreased to 25% A in 8 min, 25% A hold on 2 min, and increased to 70% A in 3 min before returning to 95% A in 1 min. The total run time was 18 min per sample. The second condition was applied by Wang et al. [69] with slight modification. The Platinum™ C18-EPS Rocket™ (53 × 7mm 3μm) (Alltech^®^, Missouri, United States) was used for reverse-phase column chromatography. The mobile phase consisted of acetonitrile and water with a ratio of 13:87. The injection volume for all samples was 10 μL. Compounds were monitored at 280 nm at a flow rate of 1 mL/mins. All determinations were performed in triplicate. Chromatograms were recorded by photodiode array detection at 280 nm. The calibration standards were prepared by serial dilutions of different polyphenols to obtain the concentrations between 25–50 μg/mL (Figures S1 and S2).

### 3.9. Characterisation of the Methanolic Fraction on Quadrupole Time-of-Flight Mass Spectrometer (QTOF-MS)

The methanolic fractions of CF confirmed the presence of the polyphenolics by QTOF-MS coupled with ZORBAX Eclipse Plus C18 (2.1 × 150 mm, 1.8 µm) and UV–Vis detector (Agilent Tech., Santa Clara, CA, USA). The sample preparation and cleaning up steps were according to Arjin et al. [49]. The instrument settings were the specific protocol for the polyphenol detection using the UV at 330 nm; 0.2 mL/min flow rate; injection volume of 10 μL. The mobile phase gradients comprised of 5% ACN and 95% water (1% formic acid), decreasing to 20% ACN in 5 min, 30% ACN in 5 min, 35% ACN in 5 min, 45% ACN in 5 min, 75% ACN in 5 min, and 95% ACN until the run ended. The MS conditions involved an electrospray ionization probe in positive and negative mode [70]. The nebulizer was operated at 20 psi with 7 L/min N_2_ flow. The capillary temperature was maintained at 300 °C, while the sample flow rate was at 8 μL/min. The *m*/*z* range was 50–1000, the capillary voltage was 4500 V, and the dry heater temperature was set at 280 °C.

### 3.10. Chemometric and Statistical Analyses

All experiments were operated in at least triplicate for each test. Comparisons of the mean of differences in antifungal activities of CF extract fractions were analyzed using a one-way analysis of variance and Duncan’s Multiple Range Test. All statistical analyses were performed using the SPSS 23.0 software (SPSS Inc., Chicago, IL, USA). A *p*-value < 0.05 was considered statistically significant. The relationships between the polyphenol compositions, antioxidant and antifungal activities were analysed using Principal Component Analysis (PCA) by the XLSTAT version 2020.

## 4. Conclusions

The polyphenols from the Arabica coffee pulp powder possess high inhibitory activity against horticultural pathogens, *A. brassicicola*, *Pestalotiopsis* sp. and *P. breviseta.* It is worth highlighting that this study is the first to use plant extract to control leaf spot pathogens in coffee. It provides an alternative way to value add the coffee by-products that support sustainable development during food production.

## Figures and Tables

**Table 1:** Phytochemical compositions of coffee pulp powder.

Coffee Pulp Powder		
Compositions	Before extraction (BCF)	After extraction (ACF)
Moisture (%*w*/*w*)	5.98 ± 0.538	6.12 ± 0.02
Carbohydrate (%*w*/*w*)	62.78 ± 0.756	61.93 ± 1.45
Crude fibre (%*w*/*w*)	15.69 ± 0.66	18.27 ± 1.03
Crude protein (%*w*/*w*)	11.13 ± 0.009	12.09 ± 0.25 *
Crude fat (%*w*/*w*)	4.10 ± 0.265	1.23 ± 0.19 *
Ash (%*w*/*w*)	0.32 ± 0.009	0.36 ± 0.11
%Yield methanol extract	6.64 ± 1.911	-
%Yield dichloromethane extract	2.27 ± 0.287	-
Crude extracts		
Polyphenols (μg/g dried sample)	Methanol	Dichloromethane
Total flavonoid content	1.03 ± 0.00	n/d
Total phenolic content	0.56 ± 0.00	n/d
Antioxidant activities (%)		
ABTS	93.13 ± 0.64	56.09 ± 0.52
DPPH	99.44 ± 0.40	65.22 ± 5.32

n/a = not available; n/d = not detectable. Data are expressed as mean ± standard error, minimum *n* = 3; * Student’s *t*-test analysis of the significant difference of proximate compositions of coffee pulp powder between before and after extraction (*p* < 0.05).

**Table 2:** Polyphenol compositions of the methanolic fraction of the Arabica coffee pulp powder.

Compositions		Methanol
Flavonoid compounds (mg/g extract)	Epigallocatechin gallate	31.76 ± 0.34
	Naringin	9.63 ± 0.00
	Epicatechin	8.66 ± 0.01
	gallate	
	Catechin	2.41 ± 0.15
	Gallocatechin	0.12 ± 0.00
	gallate	
	Quercetin	5.42 ± 0.00
Non-flavonoid compound (mg/g extract)	Caffeic acid	68.05 ± 0.45
	Caffeine	21.59 ± 0.00
	P-coumaric	11.04 ± 0.02
	Rosmarinic acid	6.41 ± 0.00
	O-coumaric	6.24 ± 0.01
	Gallic acid	2.41 ± 0.15

Data are expressed as mean ± standard error, *n* = 3.

**Table 3:** Characterisation of the methanolic fraction on quadrupole time-of-flight mass spectrometer (QTOF-MS).

Polyphenol Groups	Relation Time	CAS No.	m/z Values				Formula	Compounds	Matching Score
			(M + H)+	(M + NH4)+	(M + Na)+	(M + CH3COO)−			
Flavonoid	-	989-51-5	-	-	-	-	C_22_ H_18_ O_11_	Epigallocatechin gallate	-
	-	10236-47-2	-	-	-	-	C_15_ H_12_ O_5_	Naringenin	-
	-	1257-08-5	-	-	-	-	C_22_ H_18_ O_10_	Epicatechin gallate	-
	-	154-23-4	-	-	-	-	C_15_ H_14_ O_6_	Catechin	-
	-	4233-96-9	-	-	-	-	C_22_ H_18_ O_11_	Gallocatechin gallate	-
	1.203	117-39-5	-	-	-	361.0544	C_15_ H_10_ O_7_	Quercetin	85.56
Non-flavonoid	-	331-393-5	-	-	-	-	C_9_ H_8_ O_4_	Caffeic acid	-
	-	501-98-4	-	-	-	-	C_9_ H_8_ O_3_	p-coumaric	-
	-	20283-92-5	-	-	-	-	C_18_ H_16_ O_8_	Rosmarinic acid	-
	-	614-60-8	-	-	-	-	C_9_ H_8_ O_3_	O-coumaric	-
	1.222	149-91-7	171.0288	-	193.0117	-	C_7_ H_6_ O_5_	Gallic acid	95.51
	1.288	58-08-2	195.0877	212.1126	217.0747	-	C_8_ H_10_ N_4_ O_2_	Caffeine (alkaloid)	98.03

- Not able to be detected by quadrupole time-of-flight mass spectrometry (QTOF-MS).

**Table 4:** Antifungal inhibition and morphology of the spores and mycelium.

	*Alternaria brassicicola*					*Pestalotiopsis* sp.					*Paramyrothecium breviseta*				
Extract Concentration (g/mL)	0.01	0.03	0.05	0.10	0.50	0.01	0.03	0.05	0.10	0.50	0.01	0.03	0.05	0.10	0.50
%Inhibition	41.02 ± 4.44 a	48.71 ± 1.48 ab	52.14 ± 3.08 b	45.30 ± 2.26 ab	70.94 ± 0.85 c	29.12 ± 8.09 a	35.44 ± 5.74 a	31.23 ± 8.63 a	32.63 ± 9.95 a	62.10 ± 5.57 b	37.50 ± 7.22 a	42.71 ± 2.75 a	41.66 ± 5.80 a	47.92 ± 5.21 a	78.12 ± 3.12 b
IC50	0.09					0.31					0.14				
Spore counts	8.90 ±1.5 bc × 10^−4^	1.31 ± 0.01 c × 10^−5^	4.9 ± 0.004 ac × 10^−4^	1.1 ± 0.001 a × 10^−4^	0 ± 0.0 a	n/a	n/a	n/a	n/a	n/a	9.85 ± 0.001 × 10^−5^	5.85 ± 0.004 b × 10^−5^	5.2 ± 0.0002 c × 10^−5^	6.70 ± 0.001 a × 10^−4^	0 ± 0.0 a
Spore morphology															
*Length*	15.05 ± 0.89 b	14.55 ± 1.36 b	12.85 ± 0.84 b	12.85 ± 1.00 b	0 ± 0.0 a	n/a	n/a	n/a	n/a	n/a	6.65 ± 0.10 bc	6.76 ± 0.12 c	6.37 ± 0.01 b	6.65 ± 0.10 bc	0 ± 0.0 a
*Width*	8.56 ± 0.17 c	8.51 ± 0.21 c	8.38 ± 0.19 c	7.12 ± 0.33 b	0 ± 0.0 a	n/a	n/a	n/a	n/a	n/a	1.95 ± 0.05 b	2.03 ± 0.05 b	2.22 ± 0.41 c	1.95 ± 0.45 b	0 ± 0.0 a
*Cell wall thickness*	1.03 ± 0.04 c	0.95 ± 0.04 c	0.08 ± 0.04 c	0.69 ± 0.03 b	0 ± 0.0 a	n/a	n/a	n/a	n/a	n/a	n/a	n/a	n/a	n/a	n/a
Mycelial morphology															
*Width*	4.39 ± 0.11 c	3.99 ± 0.17 bc	3.39 ± 0.15 a	3.67 ± 0.14 ab	3.88 ± 0.15 b	2.63 ± 0.11 ab	3.80 ± 0.15 c	3.01 ± 0.15 b	2.87 ± 0.14 b	2.40 ± 0.12 a	2.45 ± 0.77 a	3.09 ± 0.08 c	3.04 ± 0.01 c	2.7 ± 0.08 b	3.040 ± 0.1 c
*Cell wall thickness*	0.54 ± 0.02 d	0.53 ± 0.02 d	0.47 ± 0.03 c	0.30 ± 0.02 b	0.184 ± 0.01 a	n/a	n/a	n/a	n/a	n/a	n/a	n/a	n/a	n/a	n/a

n/a = Not available. Data are expressed as mean ± standard error, n = 30; values followed by different letter(s) in the same parameters within the same pathogen are significantly different (*p* < 0.05).

## Data Availability

Not applicable.

## Supplementary Materials

The following are available online at https://www.mdpi.com/article/10.3390/plants10071422/s1, Figure S1: Chromatograms of polyphenol standards (A); Gallic acid (1); Catechin (2); Epicatechin (3); Gallocatechin gallate (4); Epigallocatechin gallate (5); Caffeic acid (6); Epicatechin gallate (7); Naringin (8); P-coumeric acid (9); Rosmarinic acid (10); Quercetin (11); O-coumeric acid (12) and crude methanolic coffee pulp extract (B) on High-Performance Liquid Chromatography analysis (HPLC) (Shimadzu, Kyoto, Japan), Figure S2: Chromatograms of catechin standards and crude methanolic coffee pulp extract run on a C18 column by High-Performance Liquid Chromatography analysis (HPLC) (Shimadzu, Kyoto, Japan).
