# Supplementary material for: Recovery of Polyphenolic Fraction from Arabica Coffee Pulp and Its Antifungal Applications
Source: Plants (Basel). 2021 Jul 12;10(7):1422. doi: 10.3390/plants10071422 (PMC8309451; doi:10.3390/plants10071422)
Supplement: Supplementary file 1 [file plants-10-01422-s001.zip › plants-1293670-supplementary.pdf]

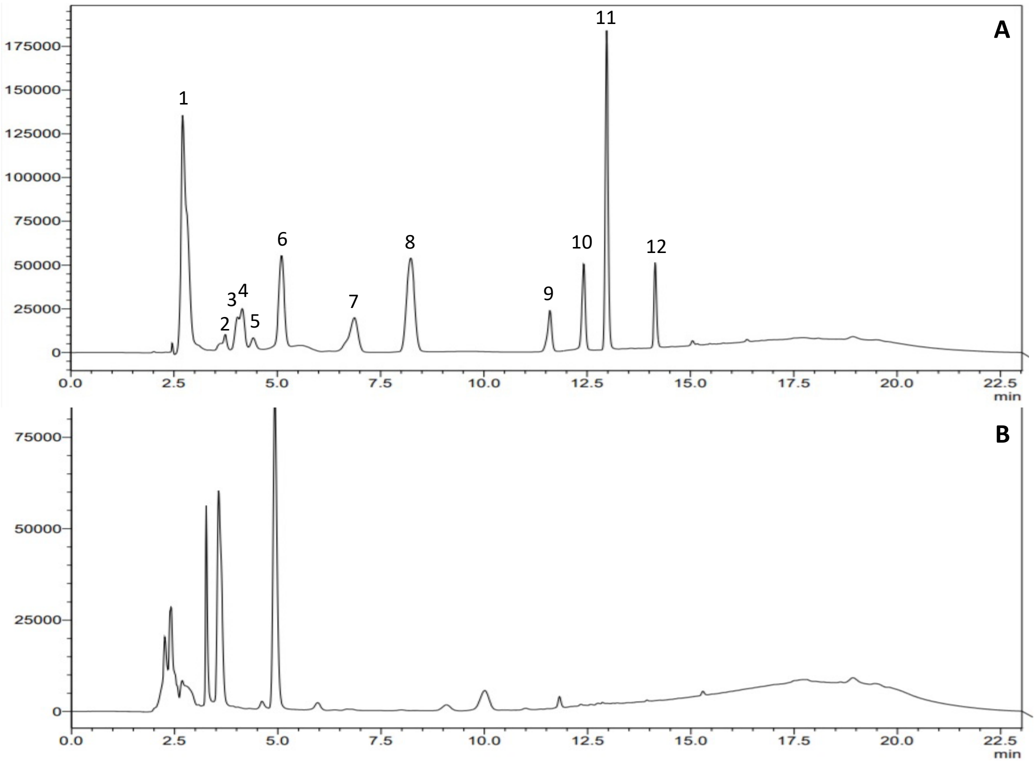

FigureS1. Chromatograms of polyphenol standards(A); Gallic acid(1); Catechin(2); Epicatechin(3); Gallocatechin gallate(4); Epigallocatechin gallate(5); Caffeic acid(6); Epicatechin gallate(7); Naringin(8); P-coumeric acid(9); Rosmarinic acid(10); Quercetin(11); O-coumeric acid(12) and crude methanolic coffee pulp extract (B) on High-Performance Liquid Chromatography analysis (HPLC) (Shimadzu, Kyoto, Japan).

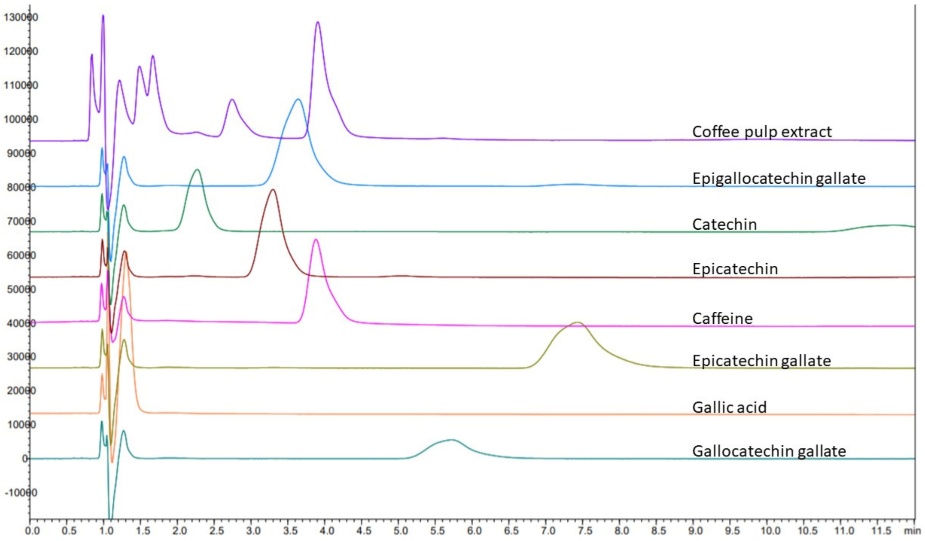

FigureS2. Chromatograms of catechin standards and crude methanolic coffee pulp extract run on C18 column by High-Performance Liquid Chromatography analysis (HPLC) (Shimadzu, Kyoto, Japan).
